# PLUG-N-HARVEST Architecture for Secure and Intelligent Management of Near-Zero Energy Buildings

**DOI:** 10.3390/s19040843

**Published:** 2019-02-18

**Authors:** Rafael Marin-Perez, Iakovos T. Michailidis, Dan Garcia-Carrillo, Christos D. Korkas, Elias B. Kosmatopoulos, Antonio Skarmeta

**Affiliations:** 1 Department of Research and Innovation, Odin Solutions, Alcantarilla, 30820 Murcia, Spain; rmarin@odins.es (R.M.-P.); dgarcia@odins.es (D.G.-C.); 2 Information Technologies Institute, Centre for Research and Technology Hellas, 57001 Thessaloniki, Greece; michaild@iti.gr (I.T.M.); chriskorkas@iti.gr (C.D.K.); kosmatop@iti.gr (E.B.K.); 3 Democritus University of Thrace, Building A Xanthi Campus Kimmeria, 67100 Xanthi, Greece

**Keywords:** IoT, ICT, wireless communications, artificial intelligence, security and privacy, energy efficiency, renewable energy, building automation

## Abstract

Building Automation (BA) is key to encourage the growth of more sustainable cities and smart homes. However, current BA systems are not able to manage new constructions based on Adaptable/Dynamic Building Envelopes (ADBE) achieving near-zero energy-efficiency. The ADBE buildings integrate Renewable Energy Sources (RES) and Envelope Retrofitting (ER) that must be managed by new BA systems based on Artificial Intelligence (AI) and Internet of Things (IoT) through secure protocols. This paper presents the PLUG-N-HARVEST architecture based on cloud AI systems and security-by-design IoT networks to manage near-zero ADBE constructions in both residential and commercial buildings. To demonstrate the PLUG-N-HARVEST architecture, three different real-world pilots have been considered in Germany, Greece and Spain. The paper describes the Spain pilot of residential buildings including the deployment of IoT wireless networks (i.e., sensors and actuators) based on Zwave technology to enable plug-and-play installations. The real-world tests showed the high efficiency of security-by-design Internet communications between building equipment and cloud management systems. Moreover, the results of cloud intelligent management demonstrate the improvements in both energy consumption and comfort conditions.

## 1. Introduction

Smart Cities [1] have emerged as the answer to cope with the demographic challenges associated with an increasingly urbanized population. As part of the integrative vision of smart cities, Building Automation (BA) is key for the development of more efficient homes and sustainable cities. In fact, according to the European Alliance of Companies for Energy Efficiency in Buildings (EuroACE [2]), we spend over 90% of our time in buildings.

Energy consumption from buildings (i.e., residential and non-residential) has steadily increased reaching levels of 40% in Europe and has overpassed the other major sectors of industry and transportation [3]. Nowadays, existing Building Automation (BA) systems are not enough for significantly reducing the energy use in the building sector, especially in high-consuming ones such as old buildings or buildings with “poor” low-cost design and construction. In fact, BA systems have the potential to achieve substantial energy savings, e.g., 31% in restaurants, 25% in hotels, 39% in offices, 49% in shopping centres, 34% in schools/universities and 27% in residential buildings [3]. For this reason, an intense activity for newer and stricter regulations for reducing the energy use in buildings is recently taking place [4]. Concretely, all new constructions will be based on Adaptable/Dynamic Building Envelopes (ADBE) [5] to achieve nearly Zero-Energy Buildings (nZEB) [6].

However, BA systems require substantial installation and operational costs in order to provide energy reductions and Renewable Energy Sources (RES) exploitation. This can be achieved by BA systems if they are able to modify the operation of the different HVAC elements and appliances of the building in an intelligent manner [7,8]. The vast majority of existing businesses and services that employ BA systems are not able to provide real-time decisions unless they use very consuming and expensive programming, with support for a fine-tuning process that runs not only during the initial deployment of the system, but throughout its lifetime. Examples of the need for a continuous calibration and tuning of the control logic are changes in weather conditions, occupants habits, building infrastructure, etc. [9,10,11]. Moreover, new BA systems must be able to intelligently manage new, more efficient constructions based on Adaptable/Dynamic Building Envelopes (ADBE). The ADBE buildings integrates Renewable Energy Sources (RES) and Envelope Retrofitting (ER) that must be managed by new BA systems based on Artificial Intelligence (AI) and Internet of Things (IoT).

Based on the ADBE concept, this paper presents the PLUG-N-HARVEST architecture based on cloud AI systems and security IoT protocols to manage near-zero ADBE constructions in both residential and commercial buildings. To demonstrate the PLUG-N-HARVEST architecture, three different real-world pilots have been considered in Germany, Greece and Spain. The paper describes the Spanish pilot of residential buildings including the deployment of IoT wireless network (i.e., sensors and actuators) based on Zwave technology [12] to enable plug-and-play installations. The real-world tests showed the high efficiency of security-by-design Internet communications between building equipment and cloud management systems. Moreover, the results of cloud intelligent management demonstrate the improvements in both energy consumption and comfort conditions.

The rest of the paper is organized as follows. Section 2 analyzes current state-of-the-art about IoT-oriented smart buildings. Section 3 presents the proposed PLUG-N-HARVEST architecture for secure intelligent management of ADBE buildings. Section 4 describes the main ICT components of the architecture deployed in the cloud. Section 5 presents several real-life pilots and the details of the first deployment in the Barcelona pilot. Section 6 describes the experimental results obtained from the main ICT components. Finally, conclusions and ongoing research are drawn in Section 7.

## 2. Related Work

After the recent advances, the building automation (BA) sector has progressed to the cloud-based era, exploiting the technologies of the micro-electronic world [13,14].

However, building automation is necessary but not a sufficient factor for an efficient building management. Building automation is the enabling factor that paves the way towards developing third party applications, which can exploit real-time data for energy efficient applications. Usually, BA-enabling platforms allow the everyday user to build an as-complex-as preferred tree of rules in an attempt to reduce user involvement in the building automation schedule. Unfortunately, such an approach may reduce human involvement, but is far from energy efficient since it relies mostly on observation from non-expert users [15].

A first attempt to fill this gap by the European as well as the international market resulted in a big ecosystem of enterprises that provide energy auditing and consultancy services, binding users to long-term contracts. Energy auditing is based on field observations, usage profiling and clustering from collected data. On the other hand, management consultancy is performed through off-the-shelf strategies, which are linked to the cluster, not tailored to the specific use case needs.

In recent years, several methodologies that enable energy management by exploiting the available building automation ecosystem have been formalized and published in the literature. Techniques utilizing modeled instances of the subject plant have emerged in recent literature, considering, “a-priori”, that an elaborate model will be available for tests [16,17,18]. As a result, such prerequisites drastically hinder their applicability to conventional, every day domestic cases where these models may not be available.

Finally, more modern variants of BA energy management systems (BA-EMS) are able to bypass the aforementioned problems through cognition systems. The main (and most popular) operational principle of these tools is based on data extraction and mining. This is similar to an identification black-box that automatically selects the strategy with the highest efficiency levels [19,20].

In an attempt to cope with the aforementioned limitations, this paper proposes a novel BA architecture implementation enabling smooth integration with a fully cloud-based, self-learning, intelligent management systems for the devices that can influence the energy efficiency as well as the energy mix at the building and district level, developed within the PLUG-N-HARVEST H2020 project (www.plug-n-harvest.eu). In Europe, almost 40% of the energy consumed in the building sector (residential and commercial) is used for heating/cooling purposes. Therefore, the ultimate goal is to automatically adjust the energy profile of individual consumers as well as synergetic districts by applying optimized control decisions on the most energy intensive devices (i.e., heat-recovery systems, existing HVACs, RES, energy storage, etc.) to reach nearly Zero Energy Building (nZEB) consumption levels.

The differentiating factor among other similar solutions is the just-enough learning performance of the built-in cognition mechanisms. Since cloud platform-providers allow on-demand (RAM use level, processing cores use level, etc.) an easy and economically affordable scale-up in the cloud is feasible for each symbiotic ICT sub-module foreseen within the proposed architecture. The main advantage of the proposed implementation is the computational lightness due to the distributed cloud topology adopted: each ICT module is performing in a plug-n-play and independent manner (no reconfiguration is necessary even in different building cases); each active part of the PLUG-N-HARVEST holistic ecosystem solution is hosted on an independent cloud-platform.

## 3. PLUG-N-HARVEST Architecture

PLUG-N-HARVEST proposes a architecture with inter-operable capabilities, flexible enough to apply to any existing (or under construction) building. This architecture aspires transforming static building envelopes to active ones by deploying off-the-shelf energy harvesting, storing and thermally enhancing elements which can dynamically adapt to the availability of exogenous energy sources. To enable maximum exploitation of the available, free environmental energy in a user and micro-climate (indoor and outdoor) oriented manner, PLUG-N-HARVEST enhances their operational capabilities by imposing intelligent ICT-based mechanisms at a building and district level for energy intensive assets management, flexibile forecasting and demand response, fault-free operation as well as secure communications. The proposed architecture is driven by the recent advances performed relative to the overarching abstract automation technologies, in the family of the Internet of Things (IoT), Energy Management and Demand Response systems (EMDRS), and protocols to provide security-by-design. The technology sector, which crosses horizontally through all ICT sub-modules foreseen herein, is Artificial Intelligence (AI). AI is used to allow control through engines that generate decisions at the building and district level. Moreover, to establish a trustworthy framework, the PLUG-N-HARVEST solution employs edge technologies to ensure its safe operation.

**Figure 1:**
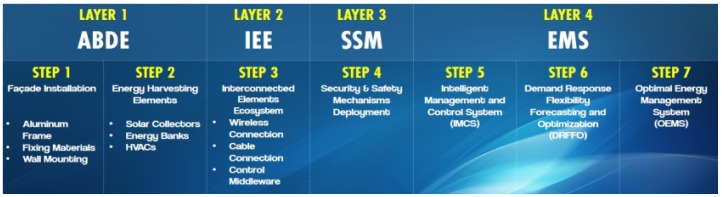
Layers of PLUG-N-HARVEST architecture.

The PLUG-N-HARVEST architecture comprises four main functional layers: Adaptive Dynamic Building Envelope (ADBE), Interconnected Elements Ecosystem (IEE), Security and Safety Mechanisms (SSM) and Energy Management Systems (EMS) (see Figure 1). The four layers provide the following functionalities:

1. Adaptive Dynamic Building Envelope (ADBE) consists in a flexible aluminum façade design with high insulation and energy-harvest elements.
2. Interconnected Elements Ecosystem (IEE) represents all hardware devices deployed at a building (or room/office) level for sensing, actuation and automation.
3. Security and Safety Mechanisms (SSM) provide authorized operation and fault detection to guarantee reliable data exchange, through:
  - Security mechanisms for advanced access control to shared data
  - Safety mechanisms for fault detection
4. Energy Management Systems (EMS) is the core AI-based cognitive adaptive optimization based on several ICT modules for closed-loop automatic control, driven by the real-time measured/sensed observations. These systems include:
  - Intelligent Management and Control System (IMCS)
  - Optimal Energy Management System (OEMS)
  - Demand Response Flexibility Forecasting and Optimization (DRFFO)

The last two layers (i.e., SSM and EMS) of the PLUG-N-HARVEST architecture integrate novel ICT components such as IoT Security/Safety mechanisms and AI-based energy management modules to achieve a secure and intelligent control solution for smart buildings. These novel ICT components and their cloud ecosystem are detailed in the next section.

## 4. Cloud Ecosystem of Integrated ICT Modules

The cloud ecosystem consists of five cooperating ICT-based elements: (a) Security Mechanisms provide the access control to sensor data and personal information; (b) Safety Mechanism enables the data fault detection; (c) Intelligent Management and Control System (IMCS) is responsible for controlling and coordinating the energy use (flow) from the energy-generating, storing and consuming elements installed at a local building level; and (d and e) the Optimal Energy Management System (OEMS) and the Demand Response Flexibility Forecasting and Optimization Tool (DRFFO) are responsible for orchestrating the energy use (flow) at a higher micro-grid level at district scale.

**Figure 2:**
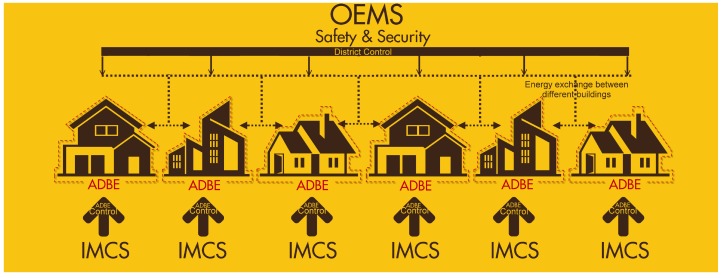
Concept of PLUG-N-HARVEST architecture.

The conceptual integration of these ICT components is shown in Figure 2. First, Security and Safety mechanisms are transverse techniques to guarantee secure M2M communications among ICT cloud-based modules and IoT networks deployed in buildings. Second, ICMS provides intelligent control at building level, while OEMS/DRFFO components provides energy optimization at district level.

In the next subsections, the security and safety mechanisms, energy management modules and their work-flow are detailed.

### 4.1. Security Mechanisms for Advanced Access Control to Sensor/Actuation Data

The PLUG-N-HARVEST architecture emphasizes the security processes to exchange large amounts of data between the different ICT systems (i.e., IMCS, DRFFO and OEMS) and the IoT equipment deployed in the buildings.

The security-by-design mechanisms of the PLUG-N-HARVEST architecture provide different security procedures to guarantee that the information and data of objects/devices connected are accessed only by those entities having the proper authorization. In particular, the integrated security-by-design mechanisms are:

- A modeling language based on XACML [21] (eXtensible Access Control Markup Language) main concepts is used to specify privacy policies on structural models describing both users and applications properties;
- A distributed access control model based on capabilities tokens (DCapBAC) [22] is provided to manage the authorization access;
- A privacy-preserving identity management solution based on KeyRock [23] protocol is integrated to the Identity Manager framework;
- A optimized elliptic curve cryptography (ECC) [24] is provided for signing and validating distributed tokens between the BMS system and external entities (i.e., constrained IoT devices and ICT system).
- A channel protection solution based on NGSI/HTTP over TLS standard (i.e., HTTPS) [25] is provided.

**Figure 3:**
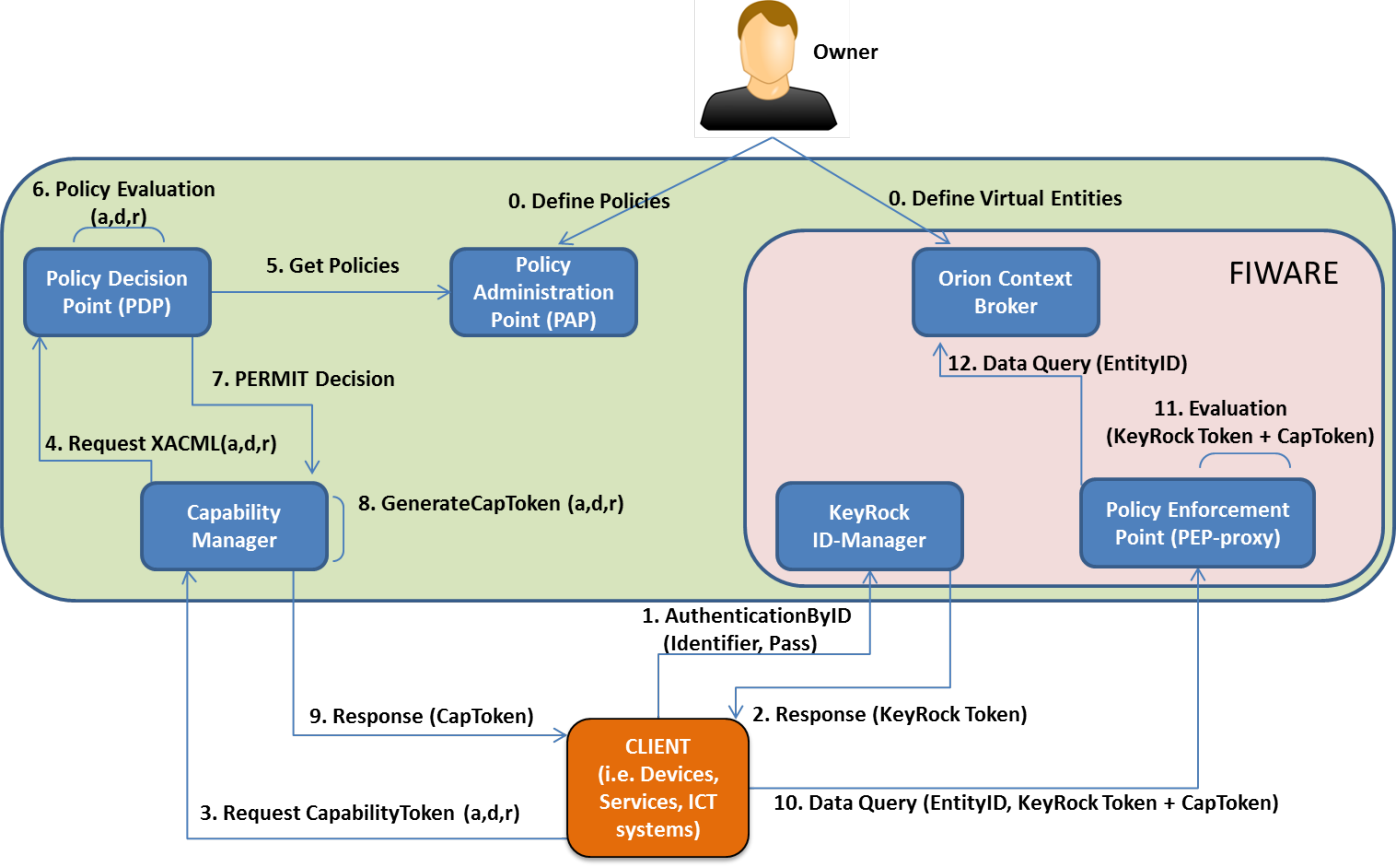
Diagram of security mechanisms integrated in BMS system.

These security mechanisms have been integrated in a Building Management System (BMS) composed of the software components shown in Figure 3:

- The Policy Administration Point (PAP) represents the point where users are empowered to define their access control policies to govern the access to the stored data.
- The Policy Decision Point (PDP) is responsible for evaluating access request against the set of access control policies that are defined in the PAP. Both PDP and PAP are based on the XACML standard.
- The Capability Manager is the component for generating DCapBAC tokens in the case of receiving affirmative authorization decisions from the PDP.
- The KeyRock ID-Manager represents the central point of the system where users, services or devices are registered, including their identifiers and attributes.
- The Orion Context Broker provides the functionality of a data broker to allow different entities to remain decoupled, by following a publish/subscribe communication based on NGSI/HTTP protocol.
- The Orion Policy Enforcement Point (PEP) Proxy is responsible for intercepting access requests to the data stored in the Orion Context Broker, by evaluating the request content in order to determine if the access must be granted or denied.

In addition to these internal components, the BMS system considers two main external entities.

- The Owner is the BMS administrator in charge of defining access control policies to guarantee only authorized clients can get access to their data.
- The Client represents a user, service or device that tries to perform a specific action (i.e., publish or retrieve) over the stored data of an entity registered in the BMS system.

Before describing the main interactions among these components, the Owner must perform the initial registration phase in the BMS system (Step 0). First, the Owner must define the virtual entities representing devices and services in the Context-Broker, which are also registered in the KeyRock ID-Manager. Second, the Owner must define the set of DCapBAC access control policies to determine which devices or services are authorized to perform which actions over their data stored in this specific virtual entity in the BMS system.After the initialization phase, when a Client (i.e., device or service) tries to perform a specific action over a registered entity in the BMS system, the Client establishes three secure HTTPS channels based on public key cryptography to communicate with the BMS components: KeyRock ID-Manager, Capability Manager and PEP proxy. Then, the Client sends an authentication request with its identifier and password registered to the KeyRock ID-Manager (Step 1). If the authentication is valid, the KeyRock ID-Manager responds with a signed KeyRock token to the Client (Step 2). Once authenticated, the Client requests a DCapBAC CapToken by querying the Capability Manager (Step 3). This query includes some attributes (i.e., action, destination, and resource). With these attributes, as well as the NGSI method and EntityID specified within the query, the Capability Manager asks the XACML-PDP (Step 4) to determine whether the requested credential must be generated. The XACML-PDP gets the policies defined by the Owner in the XACML-PAP (Step 5), and validates the policies against the query (Step 6). If the authorization is valid (Step 7), the Capability Manager generates a signed CapToken for the Client (Step 8). Once the CapToken is obtained (Step 9), the Client performs a data query (i.e., a NGSI method over the specific EntityID) including both tokens (i.e., KeyRock and CapToken) towards the PEP proxy (Step 10). The PEP proxy evaluates both tokens by validating the authenticated KeyRock token and whether the requested operation (i.e., NGSI method over the EntityID) is authorized in the CapToken (Step 11). To do that, the PEP Proxy verifies both ECC signatures of CapToken and KeyRock-token based on public key cryptography in the BMS system. If both validations are right, the PEP proxy completes the Data Query over the Orion Context Broker (Step 12). For instance, this Data Query could be to update or retrieve data from the specific EntityID (e.g., a registered IoT device). The BMS system with security mechanisms acts as the center point for the publication and subscription of all sensor and actuation data that will be exchanged among cloud energy-management systems (i.e., IMCS, OEMS, DRFFO) and IoT networks in the buildings. These security mechanisms ensure that a high-level of data protection are applied throughout the PLUG-N-HARVEST architecture. Moreover, these mechanisms empower the building owner with full control over the access and use of their data.

### 4.2. Safety Mechanism for Data Fault Detection

PLUG-N-HARVEST integrates a safety mechanism for fault detection and filtering of data traffic exchanged among building IoT networks and energy-management systems through the BMS system. This safety mechanism [26] was developed in the RERUM EU project (*ict-rerum.eu*) based on a Complex Event Processor (CEP) [27]. The Safety CEP consists of a state machine which computes the incoming events according to a set of rules defined in a declarative language (called DOLCE [28]) which is used to correlate the events that are received.

**Figure 4:**
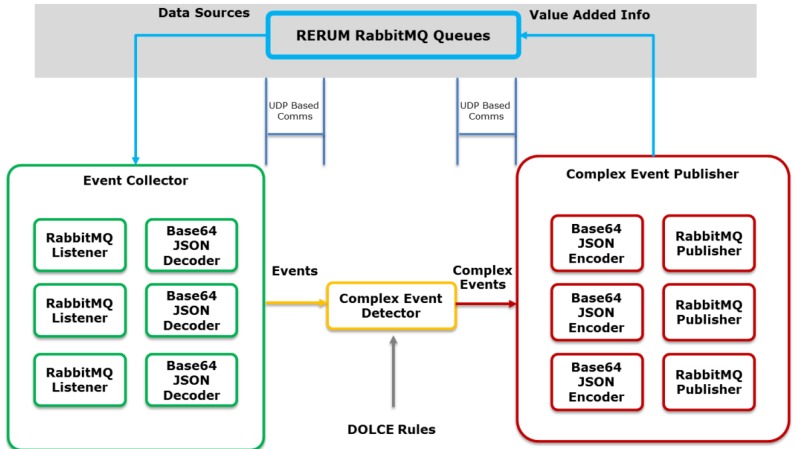
Diagram of safety mechanisms for fault detection.

Figure 4 shows the implementation of the Safety CEP engine containing three main components: Event Collector, Complex Event Detector and Complex Event Publisher. First, the Event Collector subscribes to the corresponding RabbitMQ [29] queues to retrieve the sensor data and send them to the Complex Event Detector in the appropriate internal format. The Detector processes the sensor data according to DOLCE rules to detect faults patterns and generates the outputs towards the Complex Event Publisher that generates the added value information.

The Safety CEP allows the near real-time faults detection of specific data streams in a large volume of observations according to structural and temporal relationships. The Safety CEP provides support for event:

- filtering;
- splitting;
- composition; and
- pattern detection (on a sequence of events) and matching.

This CEP mechanism enables several functionalities: (a) models for detection of repetitive false-data patterns; (b) recognition algorithms of processing patterns based on event and data flows to establish device-centric context; and (c) advanced models to mitigate the faulty data and operational patterns (i.e., “normal” system behavior) from the data flow, which includes the analysis of the data input, output and its related context.

In the PLUG-N-HARVEST architecture, the safety mechanism is integrated inside the BMS system to detect faults and filter data before the data storage in the Context Broker.

### 4.3. IMCS: Intelligent Management and Control System

**Figure 5:**
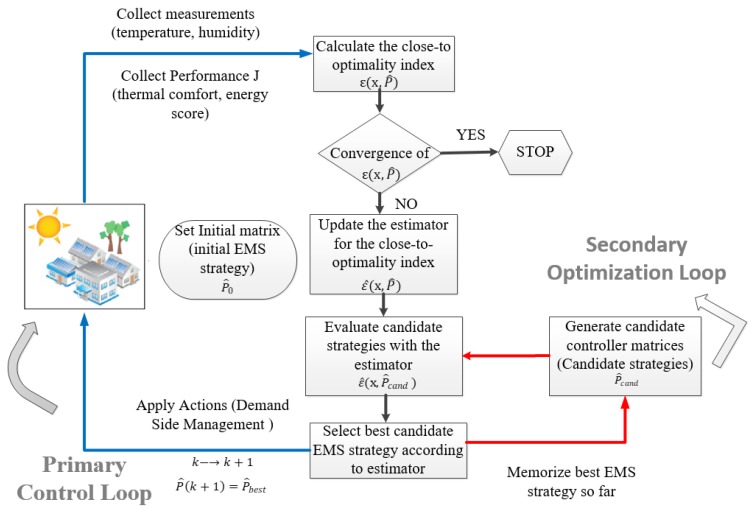
IMCS strategy description.

The IMCS is an advanced building energy management system, enhancing the “intelligence” levels of existing building management systems (BMS) and fully-exploiting data collection and actuation capabilities of the BMS in building operation mode. The IMCS supports energy management of the fully-operational building. Moreover, it can efficiently coordinate and control integrated renewable energy sources for heating, cooling and ventilating systems operating under many different protocols and provided by many different vendors with most of them being off-the-shelf components. Figure 5 depicts the IMCS procedure, which is described as follows:

- The process starts by initializing an abstract control matrix based on the real-time observed measurements and determines the control actions to be applied for the next time-step.
- Next, a linear-in-parameters estimator (LIP) [30] is constructed according to the combined performance index values, which are linked with the respective control matrix defined.
- Based on this estimator, random semi-definite positive perturbations of the control matrix are evaluated to estimate and select the one that is expected to present the best performance index.
- The selected control matrix candidate is applied, according to the Hamilton-Jacobi-Bellman optimal control equation [31] to the system for the next optimization period, while the process is repeated from the start until performance convergence is reached.

More details about the IMCS strategy can be found in [9,32].

In the PLUG-N-HARVEST architecture, IMCS is responsible for making optimal decisions based on building sensors and weather forecasting data to minimize energy consumption and maximize energy harvesting. This is achieved through two key strategies: forecasting and demand shaping. IMCS utilizes weather forecasting and occupancy patterns to predict solar availability and energy needs during the next hours. Based on that information, IMCS decides about the optimal use of solar power (spent on the building or saved on the battery), as well as about the HVAC setpoints, utilizing pre-cooling or pre-heating techniques, if energy availability is high. In this way, the control IMCS strategy makes close-to-optimal decisions aiming for lower costs not only for the present but also for the near future (e.g., fully charging the storage devices for high pricing hours or periods of low solar availability). The practical implementation of the IMCS tool became initially available in MATLAB/Simulink [33]; however, recently, C (Microsoft Corporation, C Language Specification Version 5.0, http://www.microsoft.com/) and Python (Python Software Foundation, https://www.python.org/) counterparts were developed considering also an easy to manipulate on-the-fly structured (re)configuration file in .XLS format as well as communication container built according to JSON Standard ECMA-262.

The IMCS is based on a fully-verified highly inter-operable tool—abbreviated in the literature as Parameterized Cognitive Adaptive Optimization [34]—which was extensively tested in several real-life cases, as part of the projects EU-FP7 AGILE (url: https://www.convcao.com/index.php/our-services/agile/) and EU-FP7 PEBBLE (url: https://www.convcao.com/index.php/our-services/pebble/) during the past years. On average, the IMCS tool presented performance improvements ranging 20–50% compared to well-established bench-marked control strategies. IMCS has been tested in diverse building management applications such as: (a) proactive control for solar energy exploitation in a German building [35]; (b) control calibration for automated energy-efficiency exploitation in an Israeli building [36]; and (c), odel-based and model-free control for building energy-efficient applications [9].

### 4.4. OEMS: Optimal Energy Management System

OEMS [37] was developed within the NOBEL GRID project (*nobelgrid.eu*) using the Smart Grid Architecture Model (SGAM) Framework [38] from CEN, CENELEC and ETSI. OEMS integrates novel smart grid services to enable energy flexibility markets, with enhanced demand-response schemes and active prosumer participation.

To enable the data exchange with smart grid services, OEMS includes some information models based on international standards such as Common Information Model (CIM) [39], COmpanion Specification for Energy Metering (COSEM) [40], IEC 61850 [41] for SCADA-related data-model standard and OpenADR [42] for demand-response activities. In the PLUG-N-HARVEST architecture, OEMS is a modular and scalable system at the district level enabling the coordination and cooperation between the different buildings and the different energy networks (electricity network, heating/cooling, domestic hot water, etc.). This system provides coordination of the energy flows within the district with emphasis on coordinating different energy flows locally—to not overload the grid.

OEMS enables different services to the facility managers, ESCOs and aggregators in order to control, manage and monitor the grid, keep them aware of the surplus of the energy harvested in order to coordinate buildings’ actions with the pilot buildings of the district as well as the other renewable energy source (RES) available in the district such as large RES installations. Its main features are: (i) monitoring and maintaining grid assets to forecast potential problems in the network; (ii) monitoring the grids for power quality and security; (iii) blackout and incident management for better time-response and quality service; (iv) increase quality of supply; (v) imbalance reduction thanks to the smart citizens’ involvement in demand response; (vi) flexibility to support system restoration after a fault; (vii) secure electricity supply; and (viii) power losses reduction thanks to power factor management. Moreover, OEMS allows stable and robust grid in order to mitigate management costs, replacement and maintenance of the grid in presence of large share of renewable energy systems and additional energy harvesting systems. This system includes advanced forecasting tools based on artificial intelligence (neural networks) and advanced statistical models to make reliable predictions of load and generation at the area of a neighborhood.

### 4.5. DRFFO: Demand Response Flexibility Forecasting and Optimization

The developed DR Flexibility Forecasting and Optimization (DRFFO) Tool constitutes a powerful dynamic and integrated tool for real-time building automated monitoring and control, allowing the forecasting of a building’s energy flexibility, based on extracted profiles and current contextual conditions, while further being able to coordinate operation of building’s assets in the optimal comfort and energy efficient manner. DRFFO first generates different operational states for a building based on the current one. These building states are simulated with the assistance of the respective building energy consumption and production forecasting methods. This simulation module provides the short-term dynamic behavior of the buildings. All these building states and their corresponding simulation results are evaluated according specific KPIs related to energy savings, occupants’ comfort, demand/response, etc. Moreover, on this evaluation and taking into account the thermal and visual comfort profiles of the occupants, DRFFO selects the optimal operational scenario for the respective building, which feeds the OEMS tool, in a form of device status changes. Furthermore, DRFFO, based on the simulated results extracted above, can dynamically estimate the current and the short-term future energy flexibility (both upper and lower demand operational states) of the building. The DRFFO tool is a human-centered approach, since its first priority and concern is to achieve the optimal building operation but without compromising the occupants’ comfort (both visual and thermal). The DRFFO tool can not only work at building level, but is also applicable at district level. Thus, it can support multiple buildings, where the overall district building behavior is simulated and evaluated leading to the selection of the optimal building status, as well as to the estimation of their flexibility. DRFFO constructs demand/response strategies to accomplish the respective missions, e.g., low or high demand, depending on the status of the grid. Within PLUG-N-HARVEST, the DRFFO will be utilized: (i) to generate building operational status; (ii) to predict the short-term operational state of the building (both prosumers and consumers); (iii) to simulate the operational states of the building; (iv) to evaluate the states based on the energy saving and the occupants comfort levels; (v) to select/make decisions about the optimal operational state for each building; (vi) to estimate the flexibility of the building; (vii) to estimate the operational status of the district; and (ix) to make decisions and apply demand/response strategies to district buildings.

### 4.6. Work-Flow of PLUG-N-HARVEST Architecture

**Figure 6:**
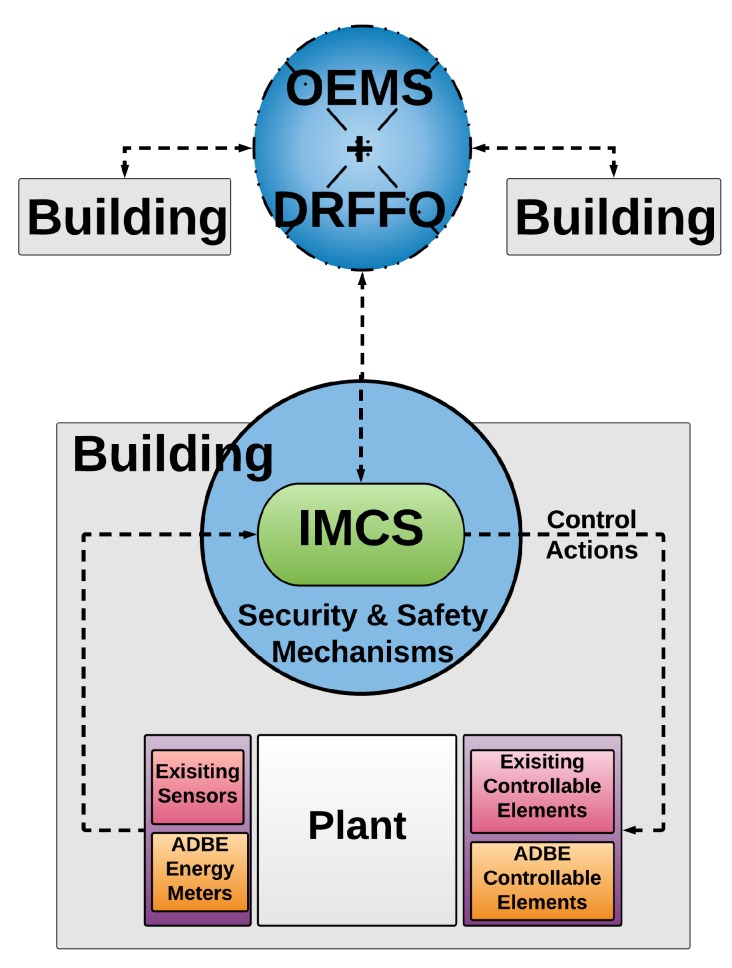
Flowchart of PLUG-N-HARVEST architecture.

The PLUG-N-HARVEST architecture employs a closed-loop building control work-flow (see Figure 6). All sensors and actuators (i.e., the ones mounted on the ADBE and home automation infrastructure) are connected the BMS system including the security and safety mechanisms. Real-time measurements collected from such building sensors together with weather data (i.e., local weather stations and online forecasting weather repositories) are accessible to energy management systems (i.e., IMCS, OEMS and DFRRO) through the BMS system. According to the sensing data, IMCS generates and applies the optimized control decisions to the building actuators (i.e., available HVACs and ADBE HVACs, RES and energy storage) through the BMS system to constitute the closed-loop building control. The control cycle frequency depends on the application characteristics: in cases where quite slow dynamics with high inertia are involved, i.e., building thermal dynamics, the control frequency is set TC = 10–15 min. The control cycle is re-calibrated periodically (e.g., in building cases on a daily basis TR = 24 h).

In this work-flow, the OEMS alongside with the DRFFO acts as energy management systems at the district and grid scale. The OEMS exploits the collected measurements in the BMS system to improve district level energy. The OEMS is a higher level module, based on a highly inter-operable and extensively evaluated approach in real-life grid (buildings-ecosystem) conditions. The OEMS preserves compatibility and co-operability with diverse communication standards for grid management. Moreover, the OEMS is responsible for orchestrating the renewable energy-shares among the interconnected buildings (district scale) utilizing the available LV power distribution grid, in an attempt to optimize the overall district energy-efficiency, fully-exploit the available green energy and result self-sufficient energy districts. DRFFO provides a novel real-time Decision Support System (DSS) constituting a powerful dynamic and integrated tool for real-time building automated monitoring and control, allowing the forecasting of a building’s energy flexibility based on extracted profiles and current contextual conditions.

PLUG-N-HARVEST creates assets and smart RES harvesting solutions that manage Distributed Energy Resources (DER) based on both distribution grid and buildings energy markets objectives. The real-time DSS takes into consideration the DER consumption prediction, which is based on DER models that use the construction elements of each space and potential DER states (operation modes and set-points). The real-time DSS gets the energy consumption prediction to make a successful flexibility forecasting at building level. This is expanded at district level for the flexibility analysis of all interconnected buildings.

### 4.7. Topology of PLUG-N-HARVEST Architecture

**Figure 7:**
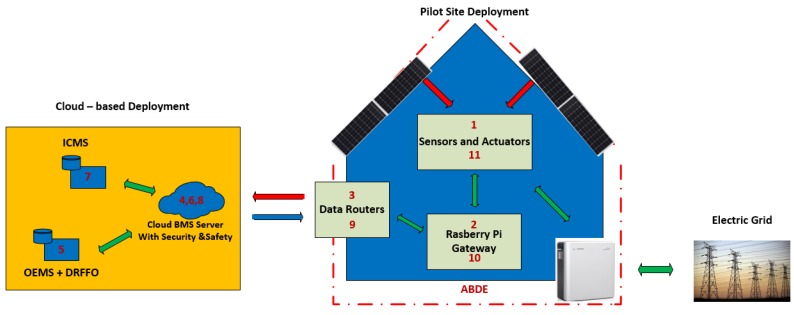
Architectural and hierarchical topology.

The architectural topology (physical and digital deployment) of all collaborating entities are shown in Figure 7. In the blue building, the physical deployment is presented, whereas the cloud digital deployment is shown in the yellow square.

The ADBE-based building deployment mainly consists of adaptive/dynamic facades, renewable energy sources (PV panels, wind turbines, etc.), storage devices (e.g., batteries) and ventilation units to enable high energy reduction and high energy harvesting. Moreover, the building deployment includes new IoT infrastructure based on wired/wireless sensors and actuators that are connected to Home Automation (HA) gateways incorporating the security protocols to communicate with the cloud BMS system through the home router. In a secure way, sensing data are sent outside the building deployment from the HA gateways to the cloud BMS system as shown in Figure 7. Through the secure BMS system, sensing data are accessible for the energy-management systems (i.e., IMCS, OEMS, and DRFFO) in cloud servers. After the data processing, these energy-management systems generate control actions that are sent back to the building, through the same routers and HA gateways to enable energy efficient actuations (HVAC systems, ventilation units, energy storage, and district transactions). To guarantee scalability and interoperability, IMCS provides the actuations at building scale while OEMS and DFRRO are integrated to provide the actuations at grid scale.

## 5. Real-Life Pilots for Demonstration

**Figure 8:**
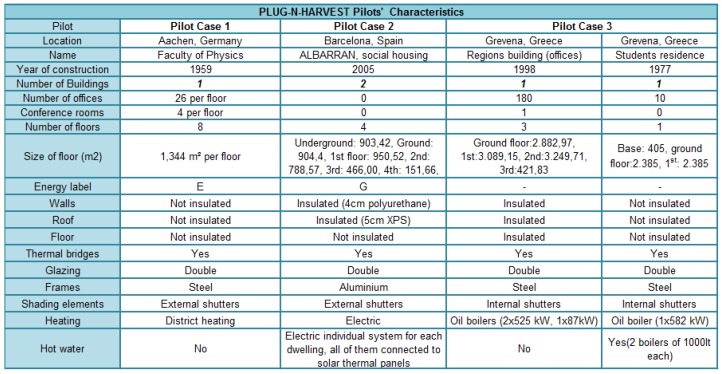
Characteristics of real-world demonstration pilots.

Three different large-scale pilots have been provided to demonstrate and evaluate the PLUG-N-HARVEST integrated system. The pilots are located in RWTH Campus, Aachen, Germany; Barcelona, Catalonia, Spain; and Region of Western Macedonia, Grevena, Greece, in order to cover different climate zones. The real-world pilots involve a varying usage pattern and both commercial (i.e., offices) and residential buildings. The main characteristics of the pilots are shown in Figure 8.

### 5.1. Objectives and KPIs

The expected benefits for residents and visitors of buildings are not limited to energy and cost savings for the buildings themselves, but also for the local municipalities where these buildings are located. Moreover, the PLUG-N-HARVEST pilots act as excellent examples for similar low energy savings municipalities throughout Europe.

The main objective of the pilots is to provide a demonstrative implementation of a smart and low cost controlling and monitoring solution for ADBE constructions to improve the energy efficiency and energy harvesting of smart houses. The pilots validate the performance of our ICT modules (i.e., ICMS/OEMS/DRFFO) operating in an integrated way to intelligently manage energy consumers and producers such as photovoltaics, electric motion, modern lighting, and other legacy heating–cooling equipment (i.e., radiators, HVAC, etc.).

In that respect, the expected research results will contribute significantly to motivating not only rich communities, but also poor communities to invest on low budget drastic energy savings and CO_2_ emission reduction strategies.

The selected pilots guarantee massive replication, after the demonstration, in low zero energy buildings and energy self-sufficient districts.

The pilots involve buildings with all different kinds of energetic, thermal and occupant interactions, and home owners/occupants of highly diverse behaviors. The overall PLUG-N-HARVEST system will be validated against the following key performance indicators (KPIs):

- Installation costs: less than 20% of the building costs.
- Operational costs: almost-zero.
- Energy bill reduction: 30–50% depending on the climate zone characteristics.
- Energy Harvesting: 50–80% primary energy reduction.
- Security/Safety User Satisfaction: 90% of the pilots users.

### 5.2. Grevena Pilot in Greece

**Figure 9:**
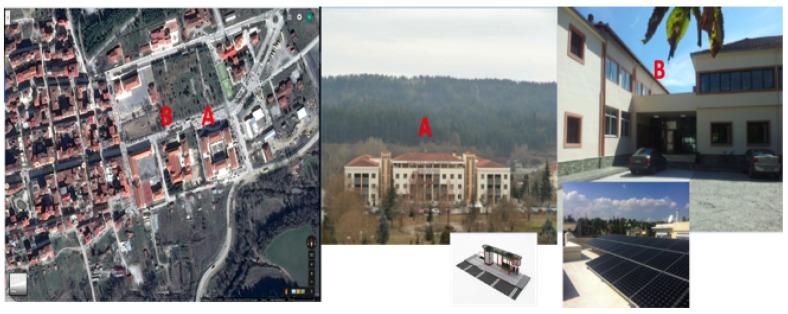
Two different buildings in Grevena pilot: (**A**) non-residential; and (**B**) residential

This pilot consists of two group of public buildings (see Figure 9) in the local Community of Grevena, Region of Western Macedonia, Greece: (A) public building of region hosts municipal authorities and municipal employees of Grevena city; and (B) residence for students hosts students of the professional school. The site consists of diversified buildings regarding the activities and users. Public building of region has a morning to noon operating and visiting hours, while the residence of students has much expanded operating hours program, even though in the morning hours the activity is lower. In the buildings, the heating system consists of oil boilers and heat pumps. Photo-voltaic system of solar panels are installed on the roof of the buildings to cover lighting consumption.

### 5.3. Aachen Pilot in Germany

**Figure 10:**
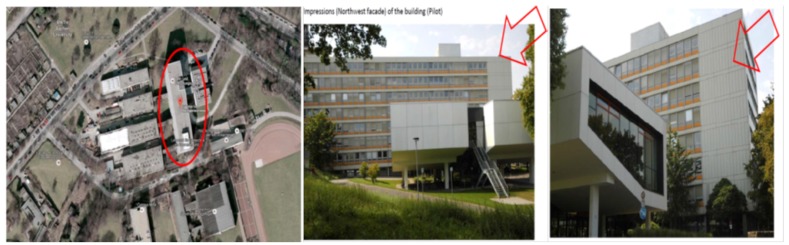
Civil Engineering Faculty in Aachen University.

This pilot is developed in the Institutional building for the Faculty of Physics in the Aachen University (see Figure 10). This pilot is a six-floor building that was built in 1959. This building has office rooms with high activity during work days. In this pilot, the heating and cooling system is provided by a co-generation unit. The offices have ventilation with heat recovery function, and the offices are heated by radiators if required. This building does not have a photovoltaic system for energy harvesting.

### 5.4. Barcelona Pilot in Spain

**Figure 11:**
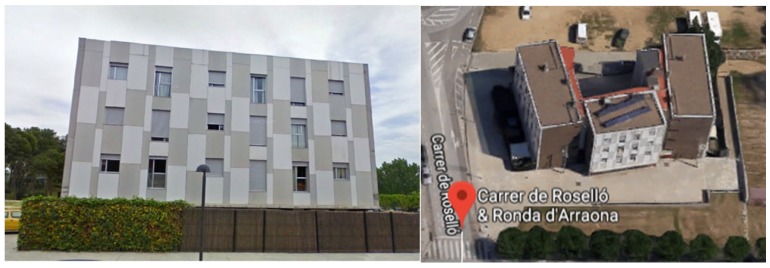
Residential site in Barcelona (Source: Habitat Agency of Catalonia).

The Barcelona pilot is focused on up to three residential buildings, as shown in Figure 11. These multi-story residential buildings are part of the same complex and belong to the Catalan Government. The pilot buildings are representative not only of the public housing stock of Catalonia region, but also of the private building stock of Catalonia. They are a multi-family building with four floors and 48 dwellings (dwellings of 1–2 bedrooms, average surface of 36–50 m^2^). Although the buildings are quite new (built in 2005), they present high primary energy consumption. In fact, they were built during the construction bubble with poor standards and low quality. In the buildings, no air-conditioning system is installed, however cable and space are reserved for the end users who could install electrical devices by themselves. The occupants currently use portable electric radiators. The power of the radiators ranges from 1000 W to 2500 W. Photovoltaic system are installed on the roof of the buildings for water heating.

### 5.5. Deployment of Barcelona Pilot

In the current stage of the PLUG-N-HARVEST project, the deployment aims to know the original building performance by collecting one-year sensors data (i.e., temperature, relative humidity, energy consumption/production, etc.) using sensing devices and Home Automation (HA) gateways connected to the secure BMS system. In the second year, it will perform the deployment of adaptive dynamic facade (ADBE) and energy efficient actuators (i.e., HVACs and ADBE HVACs, renewable energy sources and energy storage) to compare the results of high energy efficiency by energy management systems (i.e., IMCS, OEMS and DFRRO).

**Figure 12:**
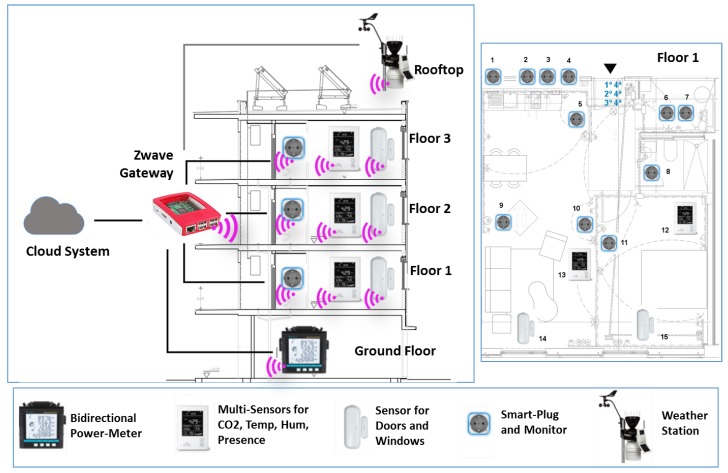
Deployment of wireless sensors and actuators in Barcelona pilot.

The first-stage deployment of wireless equipment (i.e., sensors and HA gateways) to monitor the buildings is shown in Figure 12. All deployed equipment are compatible with a recent wireless technology called Zwave [12] enabling the plug-and-play installation of battery-powered smart house products without cables. Currently, Zwave products are manufactured by 700 companies with more 2400 smart devices (i.e., sensors and actuators). The Barcelona deployment includes several commercial Zwave products for smart houses such as power-meters, multi-sensors, door sensors, smart-plug-meters, weather stations and HA gateways.

The right side represents the deployment in a building including five floors. In the building, all Zwave pieces of equipment are connected wirelessly with Zwave HA gateways providing the Internet connection with the PLUG-N-HARVEST system deployed in the cloud. The data exchange between building equipment and cloud system is performed by security-by-design protocols to protect against cyberattacks, as explained in Section 4.1.

The ground floor has the room with energy counters that require bidirectional power meters. The rooftop has solar panels for energy production and a weather station for monitoring the environmental conditions that are integrated in the PLUG-N-HARVEST system for predicting the energy production according to weather forecasting data. The building poses three main floors with two houses per floor. The left side shows a house divided into a kitchen, a bedroom, a living room and a bedroom.

Using this wireless equipment, the PLUG-N-HARVEST system enables a precise data collection from the building, as described in Section 4.

## 6. Experimental Results

In this section, we provide the results of the preliminary experiments performed thus far for the main ICT components of the PLUG-N-HARVEST architecture to provide secure and intelligent management at building scale. The real-life experiments showed the performance of the following ICT components: security mechanisms and Intelligent Management and Control System (ICMS).

### 6.1. Security Mechanisms with Building Management System (BMS)

To demonstrate the advantages of the security mechanisms of PLUG-N-HARVEST architecture on a real IoT deployment, we employed real devices for the evaluation of the secure data exchange between the BMS system in the cloud and a Home Automation (HA) scenario based on the mentioned Barcelona pilot. Concretely, we analyzed the performance of security mechanisms (i.e., KeyRock authentication, DCapBAC access control and Protected Data Exchange) in the communication between the BMS system and a HA gateway that acts as Client. To do that, we deployed the BMS system with security components (see Figure 3) on an cloud server with 2.7 GHz, 4 GB RAM and fiber-based Internet connection. Moreover, we developed a Home Automation gateway based on RaspberryPI hardware including a 1.2 GHz CPU, 1 GB RAM and an Ethernet Internet connection. The HA gateway integrates the security mechanisms to communicate with the BMS server through Internet to include an Open Source Software called HomeAssistant [43] to control wireless sensors and actuators. Concretely, we used Zwave sensors and actuators [12] such as smart-plug, energy meter, window-sensor and environment sensors. To integrate security mechanisms and HomeAssistant software in the RaspberryPI gateway, we developed a new JAVA application called SecureApp. First, this SecureApp uses the REST interface provided by HomeAssistant to manage Zwave devices in order to gather sensor data (i.e., temperature, humidity, power consumption, etc.) and request actuation operations (i.e., turn on/off). Second, the SecureApp implements the client part of three security protocols (i.e., KeyRock, DCapBAC, and HTTPS) to interact with the BMS server. Before describing the secure operations of the RaspberryPI gateway, it is assumed that the Owner has performed the initial registration phase in the BMS server. This initial phase requires the creation of a virtual entity representing the RaspberryPI gateway with its KeyRock identifier and attributes according the data provided by its connected sensors and actuators. Moreover, the Owner has defined the set of DCapBAC access control policies to determine which devices or services are authorized to perform which actions over their data stored of this specific virtual gateway in the BMS system. In particular, the Owner defines the access policies to enable the RaspberryPI gateway to publish sensor data and retrieve actuation data from its virtual entity. After the initialization phase, the RaspberryPI gateway with the SecureApp must perform the KeyRock authentication and the DCapBAC authorization. First, the RaspberryPI sends the identity credentials to the KeyRock component within the BMS system that validates the identity and provides an authentication token. Second, the RaspberryPI sends a query of data access to the DCapBAC Capability-Manager in the BMS system that validates the data access and provides an authorization token. Using the authentication and authorization tokens, the RaspberryPI can publish and retrieve data of its virtual entity in the BMS system through the PEP-Proxy. To do that, the RaspberryPI creates a secure communication channel (i.e., HTTPS) and sends both tokens that are validated by the PEP-Proxy. If the resolution is positive, the RaspberryPI can publish sensor data and retrieve actuation data of its virtual entity in the BMS system through the PEP-Proxy in a secure way. Using the same SecureApp, the energy management systems (i.e., IMCS, OEMS, and DFRRO) can establish a secure communication to interact with the BMS system to retrieve sensor data and publish actuation data to the virtual entities representing the RaspberryPI gateways. Thus, the BMS system acts a secure central point for all secure data exchange between energy management systems in the cloud and the home automation gateways in the building sites.

**Figure 13:**
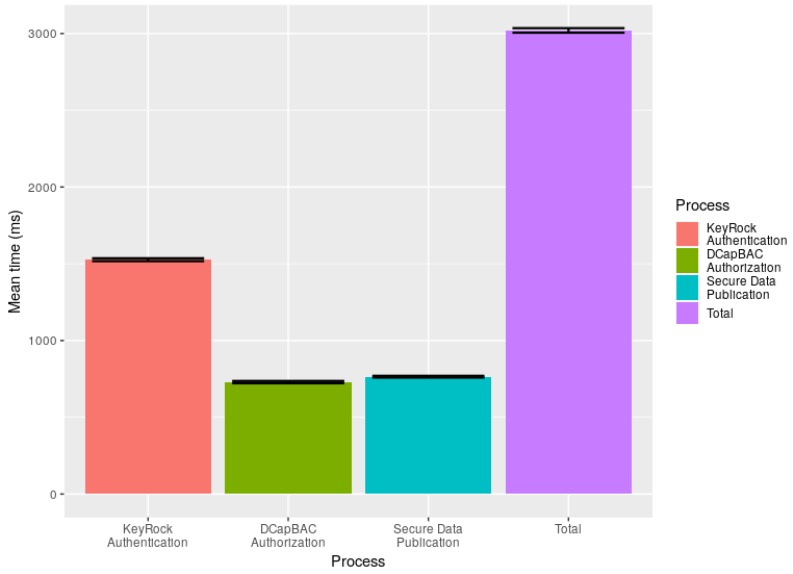
Mean time in seconds of the different processes to send information to the BMS.

Figure 13 shows the times required to perform each above-mentioned phases of the security operations between the BMS system in the cloud and the RaspberryPI gateway in the building. First, the KeyRock authentication takes approximately a mean time of 1500 milliseconds. This time entails establishing a HTTPS connection with the KeyRock ID-manager for the identity validation and gathering the authentication token. Second, the DCapBAC authorization takes approximately a mean time of 700 milliseconds. This time also entails a HTTPS connection with the Capability Manager for access policies validation and getting the authorization token. Finally, the data exchange entails another HTTPS connection with the PEP-Proxy for validating both tokens and publishing/retrieving data into the specific virtual gateway of the BMS system accordingly. This takes approximately a mean time of 750 milliseconds. Since the generation and validation of the tokens are performed in the BMS system located in the cloud, the times obtained are very efficient and consistent, as can be observed by the small confidence intervals. These three phases take approximately three seconds to complete. However, the authentication and authorization phases are only required to start the secure communication with the BMS system. Therefore, every next data exchange presenting both tokens can be performed with the BMS system in only ≈750 milliseconds.

### 6.2. Intelligent Management and Control System (IMCS)

**Figure 14:**
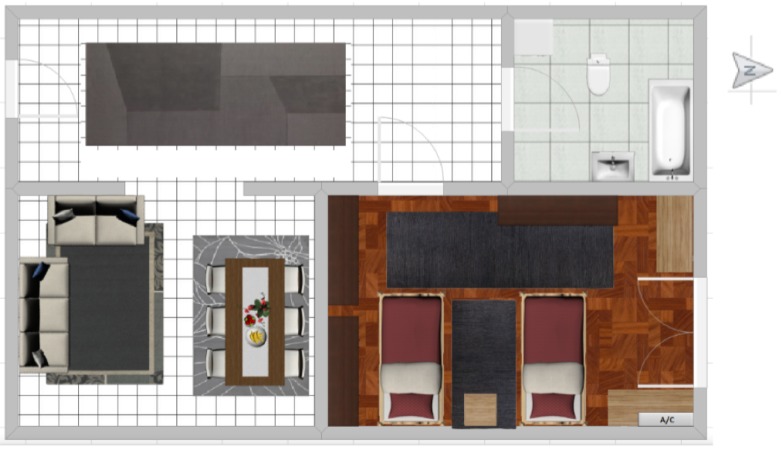
The IMCS real-life validation testbed (bottom-right room).

In the PLUG-N-HARVEST architecture, the IMCS module was verified in a small scale testbed consisted by a single room 19 m^2^ as part of a real pilot. The specific room communicates with the rest of the house through a typical indoor wooden door, which was kept closed during the tests to minimize the effects of losing heat simplify the validation of the results. Moreover, a big single-glass aluminum window is installed in the north facade of the room. On the other hand, the pilot does not have renewable energy sources (i.e., solar panel, batteries, etc.) (see Figure 14).

**Figure 15:**
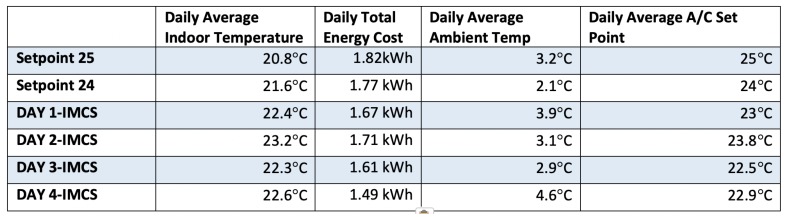
The IMCS real-life preliminary validation results.

In the pilot, a typical commercial air-conditioning (A/C), 9700 btu is used as the climate control equipment deployed next to the corner where the north and west sides of the room meet. Moreover, a RaspberryPI gateway was installed in the exact same room acting as Home Automation (HA) gateway. This HA gateway monitors the room temperature and the A/C’s consumption by a wireless smart-plug device to turn on/off the A/C equipment. Figure 15 shows the preliminary experimental results obtained during January 2019 for four consecutive days. The objective of the preliminary tests was to reduce the power load of the A/C while achieving comparable, if not better, indoor thermal comfort conditions. The observed results accounted for the efficiency of the IMCS module on optimizing the energy consumption from 1.82 kWh to 1.49 kWh within only four testing days. The IMCS outperformed both the commonly used, typical set-point strategies, which consider the A/C temperature constantly set equal to 23 and 25 degrees Celsius. According to the results, the IMCS improved both energy consumption and the comfort-penalizing index, even during colder winter days. In addition, these IMCS results will be improved in the second stage of the pilots deployment that enables the management of adaptive dynamic building envelope (ADBE) and renewable energy sources (RES) for energy harvesting.

## 7. Conclusions and Future Work

For new complex and adaptive constructions, building automation represents a crucial aspect to achieve more sustainable cities and smart homes. Accordingly, this work proposed an novel architecture for secure and intelligent management of near-zero energy constructions such as ADBE (Adaptable/Dynamic Building Envelopes). In the architecture, we proposed the integration of three cloud energy management systems (i.e., IMCS, OEMS, and DFRRO) to achieve advantages control of energy efficient operations at building and district scales. To provide secure communications between cloud systems and building infrastructure, the architecture incorporates a novel BMS system with security-by-design mechanisms. The proposed BMS system integrates five security-by-design protocols (i.e., XACML, KeyRock, DCapBAC, ECC and HTTPS) to enable authentication, authorization, optimized cryptography and channel protection in data exchanges with Home Automation (HA) gateways in the building sites. In this paper, we explain the first-phase deployment of IoT wireless networks (i.e., HA gateways, sensors and actuators) based on Zwave technology to monitor the energy consumption and the environment conditions during the first year in a Spanish pilot of residential buildings. The real-world tests showed the efficiency of security-by-design Internet communications between a Home Automation gateway and the cloud BMS server. Moreover, the preliminary results of intelligent management control system (IMCS) demonstrate the improvements in both energy consumption and comfort conditions in a real pilot. In the second phase of the deployment as future work, we will evaluate the complete performance of energy management systems (i.e., IMCS, OEMS and DFRRO) at building and district scale in the aforementioned pilots with new infrastructure (ADBE, Renewable Energy Sources, Energy Storages, etc.) that will be deployed.
